# Prevalence and reasons for use of Heated Tobacco Products (HTP) in Europe: an analysis of Eurobarometer data in 28 countries

**DOI:** 10.1016/j.lanepe.2021.100159

**Published:** 2021-07-14

**Authors:** Anthony A. Laverty, Constantine I. Vardavas, Filippos T. Filippidis

**Affiliations:** a Public Health Policy Evaluation Unit, School of Public Health, Imperial College London, United Kingdom; b Department of Oral Health Policy and Epidemiology, Harvard School of Dental Medicine, MA, USA; c School of Medicine, University of Crete, Greece

**Keywords:** Heated tobacco, tobacco control, smoking

## Abstract

**Background:**

Heated Tobacco Products (HTP) are a relatively new class of tobacco products, with limited data on usage patterns. We assessed the prevalence and reasons for use among persons aged ≥15 years in 27 European Union member states and the United Kingdom·

**Methods:**

The 2020 Eurobarometer (93·2) survey was analysed (n=28,300, aged ≥15). Multi-level regression analyses assessed socio-demographic differences in use while separate analyses investigated reasons for starting to use HTP. Results are presented as adjusted Odds Ratios (aOR) and weighted percentages with 95% Confidence Intervals (95%CI).

**Findings:**

Overall, 6·5% (95% CI 6·1;7·0) of participants had ever used a HTP. 1·3% (1·1%;1·5%) of participants were current users of HTP, and 0·7% (0·6% to 0·9%) daily users. Current and former tobacco smokers were more likely than never tobacco smokers to use HTP (aOR 36·3 (22·9;57·5), and 7·3 (4·3;12·3) respectively. Youth aged 15-24 years of age were substantially more likely to report use, e.g. aOR for ever use=7·77 (6·56;9·21) compared to those aged ≥55 years. 51·3% of ever HTP users reported at least weekly concurrent use of combustible tobacco. Among those who reported ever use of HTP, but not e-cigarettes, the most popular reason for use was the perception that HTP are less harmful than smoking tobacco (39·5%), followed by use by friends (28·4%) and stopping or reducing smoking (28·2%).

**Interpretation:**

Considerable numbers of people in the EU have ever used HTP, although current and daily use remains low. Current use is more common among younger people, and current and former smokers.

**Funding:**

None

**RESEARCH IN CONTEXT**

**Evidence before this study**

Heated Tobacco Products (HTP) are a relatively new class of tobacco product and limited data exists on their use, particularly from cross-national studies. Within Europe, there are two relevant studies: one compared levels of awareness and use between 2016 and 2018 in 6 European countries; and another from 2017/18 in 11 EU countries. These studies indicate that levels of use are low, and more common among younger people, but the potential for rapid growth means more upto date data is needed.

**Added value of this study**

6·5% of participants in the EU had ever used a HTP. 1·3% of participants were current and 0·7% were daily users. Current and former tobacco smokers were more likely to use HTP as were younger people. 51·3% of ever HTP users reported at least weekly concurrent use of combustible tobacco. Among those who reported ever use of HTP, but not e-cigarettes, the most popular reason for use was the perception that HTP are less harmful than smoking tobacco.

**Implications of all available evidence**

While current use of HTP in Europe remains low, considerable numbers of people have used them, and this is concentrated among certain groups.

Alt-text: Unlabelled box

## 1 INTRODUCTION

Heated Tobacco Products (HTP) heat tobacco to lower levels than required for ignition, but release aerosols including nicotine and other chemicals which are inhaled [1]. There are a range of products available which use different mechanisms to achieve heating, and they typically include a holder which is combined with processed tobacco in the form of sticks or capsules for use [2]. They were initially trialled in the 1980s, but were not popular with customers and were discontinued. They have recently seen a rebirth however, launched in Japan in 2014, and now all of the transnational tobacco companies have a brand of HTP. Well known versions include *iQOS* (Philip Morris International) and *glo* (British American Tobacco) [3]. In Europe they were first launched in Italy, and both Western and Eastern Europe are now considered key markets [4]. Their use has been driven in part by marketing as “reduced risk products”, which has exploited loopholes in regulation and been used by the tobacco industry to enhance their reputation and credibility [5]. A range of strategies are used to promote HTP, including claims that they are safer than traditional tobacco smoking, opening dedicated stores, using pricing strategies to attract custom, as well as social media and traditional media [6,7]. A lack of independent research into these products has been noted as an issue, and the European Respiratory Society notes concerns about possible gateway impacts and the harmful nature of all tobacco in its position statement on the issue [8].

The recent advance of HTP has been rapid with 28·3% growth in the market in 2019 [4]. In 2021 Japan is predicted to be the largest HTP market by market share, with seven of the top ten countries being in Europe (Turkey, Slovakia, Portugal, Poland, Germany, Sweden, Italy) [9]. Nonetheless there is a lack of data on prevalence and reasons for use in Europe, with the exception of a small number of studies conducted soon after the introduction of HTP into the European tobacco market. For example, analyses in six European countries between 2016 and 2018 found an increase in awareness and use between these time points, albeit based on low figures at baseline [10]. Further cross-sectional analyses from 2017/18 in 11 EU countries found that 1·8% of people had ever tried HTP; a figure which was higher among smokers and e-cigarette users [11]. This lack of data is a concern as the experience of e-cigarettes highlights the potential for the rapid growth of certain market segments within short timeframes [12,13]. When the current Tobacco Products Directive (TPD) was enacted HTP were not a part of the European tobacco market. They were classified under the broad umbrella of “novel tobacco products” and are under less stringent regulation than tobacco and e-cigarettes. However, as updates to the Tobacco Tax Directive are planned and in light of the upcoming assessment report of the TPD (which is mandated to be completed in 2021), policymakers would benefit from the provision of adjusted analyses to inform evidence based policies [14]. This study adds to the evidence base by presenting comparable, representative data across 28 nations of the EU on the prevalence and reasons for HTP use.

## 2 METHODS

### 2.1 Data source

We analysed data from all 27 European Union Member States and the United Kingdom (UK) collected in August-September 2020 through Eurobarometer survey 93·2. The standard design of Eurobarometer surveys is based on multi-stage sampling in which primary sampling units (PSU) are selected from each region within each country, proportional to population size. Within each PSU, starting addresses are selected randomly and a standard random route is followed to systematically select participating households. One randomly selected person in each household is interviewed face-to-face in the local language. However, due to restrictions for the COVID-19 pandemic, interviews were conducted online in six countries (Estonia, Finland, Ireland, Luxembourg, Sweden, United Kingdom), while data were collected through a mix of online and face-to-face interviews in four countries (Belgium, Denmark, Spain, Netherlands). In all cases, the online samples were selected through a probabilistic design [15]. Post-stratification and population size weights included in the Eurobarometer dataset ensure that samples are nationally representative in terms of age, sex, and area of residence. A total of 28,300 individuals aged 15 years and above were interviewed across the 28 participating countries (Suppl. Table 2).

### 2.2 Measures

#### 2.2.1 Tobacco smoking

All individuals were asked “Regarding smoking cigarettes, cigars or a pipe, which of the following applies to you?”. Responses included “You currently smoke” (current smokers); “You used to smoke but you have stopped” (former smokers); and “You have never smoked” (never smokers).

#### 2.2.2 Heated tobacco products

All participants were asked “Thinking about the following products [heated tobacco products], which of the following applies to you?”. Responses included “You currently use it”; “You used to use it but you have stopped”; “You have tried only once or twice”; “You have never used it”; “Don't Know”. Individuals who responded that they currently use HTP were considered current users. All those who said that they use it currently, in the past, or have only tried it once or twice were considered ever users of HTP. Current HTP users were further asked how often they use HTP (every day; every week; every month; less than monthly; you have tried only once or twice; never). We classified those who responded “every day” as daily HTP users.

#### 2.2.3 Other tobacco and related products

Participants were also asked similar questions about their experience and frequency of use of a range of tobacco and related products, including combustible tobacco (boxed cigarettes; hand-rolled cigarettes; cigarillos; cigars; pipe; and waterpipe), smokeless tobacco (oral; chewing; or nasal tobacco) and e-cigarettes (with and without nicotine). In our analysis, we refer to combustible tobacco and smokeless tobacco as tobacco products. We also refer to all tobacco products and e-cigarettes with nicotine as nicotine products.

#### 2.2.4 Reasons for heated tobacco products use

Individuals who had used HTP (but not e-cigarettes) were asked to report factors that were important in their decision to start using HTP. Respondents could select up to three of the following options: “to stop or reduce tobacco smoking”; “they were cool or attractive”; “you could consume tobacco in places where tobacco smoking was not allowed”; “they were cheaper than other tobacco products”; “your friends used heated tobacco products”; “you liked the flavours of heated tobacco products”; “you believed that these products were less harmful than smoking tobacco products”.

#### 2.2.5 Socio-demographic data

The survey collected data on age (15-24; 25-39; 40-54; and ≥55 years), sex (male; female), education: (up to lower secondary; upper secondary; tertiary up to bachelor; masters degree or above), difficulties to paying bills during the last twelve months (almost never/never; and from time to time/most of the time) and area of residence (rural; and urban).

### 2.3 Statistical analyses

We conducted multivariable logistic regression analyses fitting two-level random intercept models, which accounted for clustering of observations within countries. All models were adjusted for sex, age, difficulty paying bills, area of residence, education, and tobacco smoking as independent variables. The first set of models, with outcomes ever, current and daily HTP use were fitted among the entire analytic sample. The second set of models, with outcomes factors that were reported as important to start using HTP were fitted among those who had used HTP but not e-cigarettes. We have used the official Eurobarometer weights for descriptive analyses to account for the sampling designs. For descriptive analyses, we used the survey-specific commands in Stata (svy) to account for the sampling design. In this analytical approach, each observation is weighted based on the official Eurobarometer weights to produce estimates that are representative of the population and consider population size and non-response. Regression analyses were conducted among cases with no missing values in any of the outcome variables or covariates. Results are presented as weighted percentages or adjusted Odds Ratios (OR) with 95% CI. All analyses were conducted using Stata 15·0 (StataCorp LLC, College Station, TX).

### 2.4 Role of the funding source

There was no specific funding for this work

## 3 RESULTS

Analytic sample characteristics are shown in Suppl. Table 2. The prevalence of ever, current and daily use varied widely among European countries. A total of 6·5% (95% CI: 6·1 – 7·0) of individuals 15 years or older across the 28 countries had ever used HTP in August-September 2020. The prevalence of current (1·3%; 95% CI: 1·1 – 1·5) and daily use (0·7%; 95% CI: 0·6 – 0·9%) were lower (Table 1). Prevalence of ever HTP use ranged from 2·8% in France to 14·6% in the Czech Republic with a total of seven countries reporting prevalence above 10%. The countries with the lowest prevalence of current and daily HTP use were Denmark (0·3% and 0·1% respectively) and Sweden (0·4% and 0·1%), while those with the highest prevalence were Czech Republic (3·1% current and 2·5% daily use) and Cyprus (3·1% and 2·3% respectively). Country-level differences in the proportions of ever users who are also regular users are also apparent between countries.

**Table 1:** Prevalence of ever, current and daily use of heated tobacco products in 28 European countries in 2020. (n=27,786).

	Ever useWeighted % (95% CI)	Current useWeighted % (95% CI)	Daily useWeighted % (95% CI)
Austria	12·0 (10·1 - 14·3)	2·0 (1·3 - 3·0)	0·6 (0·2 - 1·4)
Belgium	7·8 (5·8 - 10·4)	1·7 (0·8 - 3·6)	0·3 (0·1 - 1·3)
Bulgaria	12·0 (10·1 - 14·1)	2·4 (1·6 - 3·5)	1·6 (1·0 - 2·5)
Croatia	6·8 (5·5 - 8·5)	0·7 (0·4 - 1·5)	0·6 (0·3 - 1·3)
Cyprus	8·2 (5·9 - 11·5)	3·1 (1·7 - 5·4)	2·3 (1·1 - 4·4)
Czech Republic	14·6 (12·4 - 17·2)	3·1 (2·2 - 4·4)	2·5 (1·7 - 3·8)
Denmark	6·0 (4·2 - 8·6)	0·3 (0·1 - 1·4)	0·1 (0·0 - 0·4)
Estonia	8·2 (6·5 - 10·2)	1·1 (0·6 - 2·0)	0·7 (0·3 - 1·5)
Finland	9·5 (5·0 - 17·3)	1·3 (0·6 - 2·8)	0·9 (0·3 - 2·3)
France	2·8 (1·8 - 4·2)	0·8 (0·3 - 1·8)	0·1 (0·0 - 0·9)
Germany	5·5 (4·4 - 7·0)	0·6 (0·3 - 1·2)	0·2 (0·1 - 0·7)
Greece	9·0 (7·4 - 10·9)	1·9 (1·3 - 3·0)	1·5 (0·9 - 2·5)
Hungary	4·8 (3·5 - 6·6)	1·3 (0·7 - 2·5)	0·9 (0·4 - 1·9)
Ireland	12·3 (10·4 - 14·5)	1·8 (1·2 - 2·8)	0·7 (0·3 - 1·3)
Italy	9·7 (8·0 - 11·8)	3·0 (2·1 - 4·3)	2·2 (1·4 - 3·3)
Latvia	13·8 (11·6 - 16·5)	2·9 (1·9 - 4·3)	2·0 (1·2 - 3·2)
Lithuania	10·6 (8·4 - 13·2)	2·2 (1·3 - 3·7)	1·7 (0·9 - 3·1)
Luxembourg	11·4 (8·2 - 15·6)	0·6 (0·2 - 1·5)	0·3 (0·1 - 1·2)
Malta	3·9 (2·1 - 7·1)	1·8 (0·7 - 4·2)	0·5 (0·1 - 2·3)
Netherlands	4·1 (2·7 - 6·3)	0·5 (0·2 - 1·1)	0·2 (0·1 - 0·6)
Poland	3·8 (2·6 - 5·4)	1·0 (0·5 - 2·0)	0·9 (0·4 - 1·8)
Portugal	7·9 (6·3 - 9·7)	1·0 (0·6 - 1·8)	0·8 (0·4 - 1·6)
Romania	5·4 (4·2 - 6·9)	0·5 (0·2 - 1·2)	0·4 (0·1 - 1·0)
Slovakia	9·6 (7·7 - 11·9)	2·5 (1·6 - 3·9)	2·1 (1·3 - 3·5)
Slovenia	7·4 (5·8 - 9·4)	1·1 (0·6 - 2·1)	0·6 (0·3 - 1·3)
Spain	6·3 (4·8 - 8·1)	1·0 (0·5 - 1·8)	0·5 (0·2 - 1·1)
Sweden	6·6 (5·1 - 8·5)	0·4 (0·1 - 1·0)	0·1 (0·0 - 0·4)
United Kingdom	6·6 (5·1 - 8·6)	0·9 (0·4 - 1·8)	0·4 (0·1 - 1·0)
**Total %**	**6·5 (6·1 – 7·0)**	**1·3 (1·1 – 1·5)**	**0·7 (0·6 – 0·9)**
**Total (N)**	**2050**	**377**	**232**

Multivariable models fit separately for each of the three outcomes found that among individuals aged ≥15 years, age was inversely associated with ever, current and daily use (Table 2). For instance, people 15-24 years of age were substantially more likely to report ever (OR=7·77; 95% CI: 6·56 – 9·21), current (3·52; 2·41 – 5·13) and daily HTP use (3·75; 2·26 – 6·23) compared to those aged ≥55 years. Current and former tobacco smokers were more likely to use HTP. We found a strong association between tobacco smoking and ever HTP use (OR=12·22 for current vs. never smokers and OR=5·58 for former vs. never smokers). However, this was even stronger for current (OR=36·30 and OR=7·30 respectively) and daily HTP use (OR=88·10 and 22·53 respectively). Compared to those with lower educational level, people who had completed upper secondary or higher education were also more likely to report ever, current and daily HTP. Being male (compared to female), living in urban areas (compared to rural) and reporting difficulties paying bills were all associated with higher odds of ever HTP use, but not current and daily HTP use.

**Table 2:** Sociodemographic factors associated with use of heated tobacco products in 28 European countries, in 2020 (n=27,786).

	Ever use		Current use		Daily use	
	%	OR (95% CI)	%	OR (95% CI)	%	OR (95% CI)
Age						
55+ years (ref)	3·0	1·00	0·6	1·00	0·3	1·00
40-54 years	6·5	2·18 (1·89 - 2·52)	1·4	1·71 (1·26 - 2·33)	1·0	2·12 (1·42 - 3·17)
25-39 years	10·5	4·09 (3·57 - 4·70)	1·9	2·68 (1·99 - 3·59)	0·9	2·94 (1·99 - 4·35)
15-24 years	10·6	7·77 (6·56 - 9·21)	1·8	3·52 (2·41 - 5·13)	1·1	3·75 (2·26 - 6·23)
Sex						
Female (ref)	5·1	1·00	1·1	1·00	0·6	1·00
Male	8·0	1·31 (1·18 - 1·44)	1·5	0·84 (0·68 - 1·03)	0·8	0·77 (0·59 - 1·01)
Difficulty paying bills						
Never/almost never (ref)	5·2	1·00	1·0	1·00	0·5	1·00
From time to time/most of the time	9·9	1·25 (1·12 - 1·40)	1·9	1·07 (0·85 - 1·34)	1·3	1·16 (0·87 - 1·56)
Education						
Lower secondary or lower (ref)	5·0	1·00	0·7	1·00	0·4	1·00
Upper secondary	7·3	1·31 (1·13 - 1·51)	1·5	1·44 (1·03 - 2·01)	0·9	1·77 (1·11 - 2·81)
Tertiary up to bachelor	8·3	1·69 (1·42 – 2·00)	1·8	2·37 (1·63 - 3·45)	0·9	3·65 (2·19 - 6·09)
Masters or above	5·5	1·43 (1·17 - 1·75)	1·0	2·14 (1·39 - 3·29)	0·7	3·31 (1·88 - 5·83)
Area of residence						
Rural (ref)	3·9	1·00	0·6	1·00	0·4	1·00
Urban	7·7	1·52 (1·36 - 1·71)	1·5	1·27 (0·99 - 1·63)	0·9	1·17 (0·85 - 1·60)
Tobacco smoking						
Never smoker (ref)	2·1	1·00	0·2	1·00	<0·1	1·00
Current smoker	16·3	12·22 (10·62 - 14·07)	4·2	36·30 (22·92 - 57·49)	2·6	88·10 (36·05 - 215·34)
Former smoker	7·2	5·58 (4·78 - 6·53)	0·6	7·30 (4·33 - 12·30)	0·5	22·53 (8·81 - 57·62)

OR= adjusted Odds Ratios from multilevel logistic regression models.

**Figure 1:**
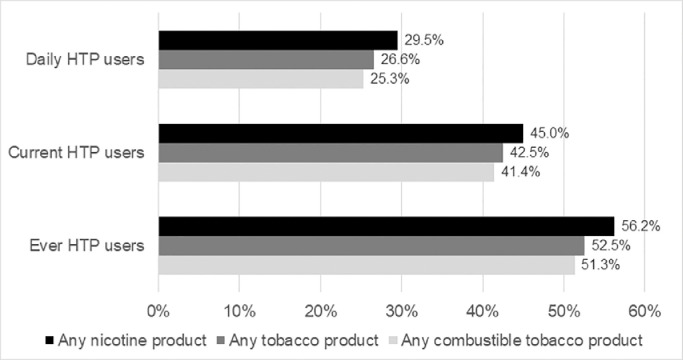
Proportion of heated tobacco products users who concurrently use other nicotine products at least weekly. Any combustible tobacco products include boxed cigarettes; hand-rolled cigarettes; cigarillos; cigars; pipe; and waterpipe. Any tobacco products include boxed cigarettes; hand-rolled cigarettes; cigarillos; cigars; pipe; waterpipe; and oral tobacco. Any nicotine products include boxed cigarettes; hand-rolled cigarettes; cigarillos; cigars; pipe; waterpipe; oral tobacco; and e-cigarettes containing nicotine.

More than half of ever HTP users reported at least weekly use of combustible tobacco at the time of the survey (51·3%; 95% CI: 47·5 – 55·0) with 56·2% reporting at least weekly use of one or more other products containing nicotine (52·4 – 59·9). These percentages were lower among current HTP users (41·4% and 45·0% respectively) and daily HTP users (25·3% and 29·5% respectively). The most frequently reported products among all three groups of HTP users were boxed and hand-rolled cigarettes and e-cigarettes (Figure 1, Suppl. Table 3).

**Figure 2:**
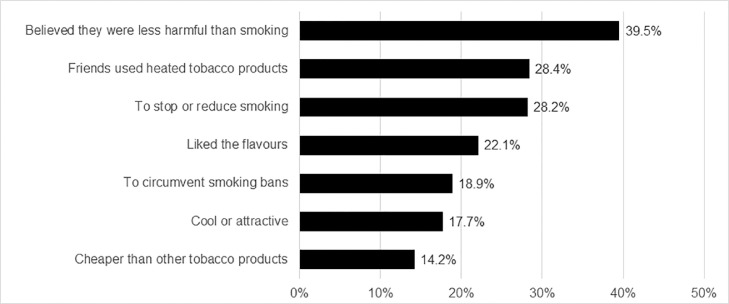
Reasons to use heated tobacco products in 28 European countries, 2020. Note: Percentages shown among respondents who ‘use or used heated tobacco products’, but not e-cigarettes (n=460).

In multivariable models among those who reported using or having used HTP, but not e-cigarettes, the most popular reason to use HTP was the perception that HTP are less harmful than smoking tobacco (39·5%), followed by use by friends (28·4%) and stopping or reducing smoking (28·2%) (Figure 2, Suppl. Table 4). Users 15-24 years old were more likely to report that they started using HTP because they were cool and attractive, to avoid smoking bans, because their friends used them and because they were cheaper than other tobacco products. HTP users with higher educational level were also more likely to start HTP to avoid smoking bans. Other sociodemographic factors were largely not associated with reporting any of the reasons assessed (Table 3).

**Table 3:** Sociodemographic factors associated with reasons to start using heated tobacco products (n=457).

	To quitOR (95% CI)	Cool/attractiveOR (95% CI)	To avoid smoking bansOR (95% CI)	CheaperOR (95% CI)	FriendsOR (95% CI)	FlavoursOR (95% CI)	Less harmfulOR (95% CI)
Age							
55+ years (ref)	1·00	1·00	1·00	1·00	1·00	1·00	1·00
40-54 years	1·09 (0·61 - 1·96)	1·96 (0·89 - 4·28)	2·09 (0·99 - 4·43)	0·89 (0·30 - 2·65)	0·76 (0·38 - 1·52)	0·67 (0·32 - 1·38)	0·59 (0·32 - 1·06)
25-39 years	0·84 (0·47 - 1·50)	1·80 (0·83 - 3·90)	1·70 (0·81 - 3·57)	2·49 (0·96 - 6·42)	1·03 (0·54 - 1·98)	1·31 (0·68 - 2·51)	0·83 (0·47 - 1·45)
15-24 years	0·57 (0·27 - 1·22)	2·63 (1·10 - 6·32)	2·38 (1·00 - 5·65)	2·82 (0·96 - 8·29)	3·71 (1·77 - 7·77)	1·78 (0·83 - 3·79)	0·69 (0·34 - 1·39)
Sex							
Female (ref)	1·00	1·00	1·00	1·00	1·00	1·00	1·00
Male	1·06 (0·69 - 1·62)	1·40 (0·84 - 2·32)	0·88 (0·54 - 1·43)	1·24 (0·64 - 2·44)	0·97 (0·61 - 1·55)	1·08 (0·67 - 1·74)	0·90 (0·60 - 1·35)
Difficulty paying bills							
Never/almost never (ref)	1·00	1·00	1·00	1·00	1·00	1·00	1·00
From time to time/most of the time	0·79 (0·50 - 1·26)	1·36 (0·81 - 2·29)	1·40 (0·84 - 2·34)	1·31 (0·65 - 2·63)	0·80 (0·49 - 1·32)	1·30 (0·80 - 2·13)	1·20 (0·77 - 1·85)
Education							
Lower secondary or lower (ref)	1·00	1·00	1·00	1·00	1·00	1·00	1·00
Upper secondary	1·16 (0·59 - 2·26)	1·99 (0·83 - 4·78)	3·11 (1·22 - 7·94)	0·40 (0·17 - 0·96)	0·96 (0·46 – 2·00)	1·61 (0·75 - 3·47)	1·87 (0·97 - 3·62)
Tertiary up to bachelor	1·16 (0·55 - 2·45)	2·11 (0·81 - 5·49)	2·80 (1·01 - 7·76)	0·51 (0·19 - 1·38)	1·98 (0·87 - 4·50)	1·64 (0·71 - 3·82)	1·74 (0·83 - 3·64)
Masters or above	1·71 (0·74 - 3·95)	1·28 (0·40 - 4·13)	3·29 (1·06 - 10·16)	0·33 (0·09 - 1·25)	1·38 (0·54 - 3·56)	0·69 (0·23 - 2·13)	1·87 (0·81 - 4·31)
Area of residence							
Rural (ref)	1·00	1·00	1·00	1·00	1·00	1·00	1·00
Urban	1·15 (0·71 - 1·87)	1·35 (0·74 - 2·46)	1·48 (0·81 - 2·69)	0·79 (0·36 - 1·75)	0·90 (0·53 - 1·52)	1·23 (0·70 - 2·17)	0·76 (0·47 - 1·21)
Smoking							
Current smoker (ref)	1·00	1·00	1·00	1·00	1·00	1·00	1·00
Former smoker	0·61 (0·38 – 1·00)	0·84 (0·48 – 1·50)	1·02 (0·59 – 1·77)	0·81 (0·37 – 1·78)	0·43 (0·24 – 0·75)	1·09 (0·64 – 1·84)	0·55 (0·34 – 0·89)
Never smoker	0·24 (0·68 – 0·86)	1·04 (0·35 – 3·04)	0·29 (0·06 – 1·32)	2·13 (0·64 – 7·07)	0·64 (0·23 – 1·80)	0·95 (0·33 – 2·75)	0·55 (0·21 – 1·44)

OR= adjusted Odds Ratios from multilevel logistic regression models.

All analyses are among respondents who ‘use or used heated tobacco products’, but not e-cigarettes.

## 4 DISCUSSION

This analysis of recent data from 27 EU member states and the UK finds that levels of current and daily use of HTP were low in 2020, although substantial numbers of people have experimented with these products. Use of HTP was more common among younger people as well as former or current smokers, and the most common reason for use was the perception that they were less harmful than other tobacco.

We found that the majority of use of HTP was among former and current tobacco smokers, which is in line with other HTP evidence as well as that from early in the growth of e-cigarettes [11,16]. HTP are marketed in some corners as being aids to stopping or reducing smoking tobacco, and if this were to be true, they would likely be positive for population health. We did find that almost one in three users of HTP did so to stop or reduce smoking, although the cross-sectional nature of this study means that we cannot ascertain if these attempts were succesful. Other emerging evidence is also ambiguous on this point, and the recent emergence of HTP precludes long-term follow up. On the contrary, cross-sectional evidence from South Korea has indicated a reduction in quitting among HTP users, while ecological analyses have pointed to a decline in visits to stop smoking clinics and a slowing in reductions of smoking rates [17,18]. Longitudinal research in Hong Kong has similarly not identified an increase in quit rates among HTP users [19].

Additionally, our findings indicate that 2·1% of never smokers have tried HTP, which given the large population of the EU would correspond to more than 9 million people. This group of people do not have previous experience with regular use of nicotine or tobacco which substantiates claims that we should be concerned about HTP should they draw more people into using tobacco. We found that substantial proportions of HTP users of all frequencies were also using other tobacco products, with for example over half of ever HTP users also using some form of combustible tobacco, as are around a quarter of daily HTP users. Other research in the US and elsewhere has indicated greater tobacco use among HTP users [20]. These levels of dual use, coupled with the fact that almost one in five HTP users reported using them to circumvent smoking bans is likely to complicate future tobacco control efforts. Similarly, research among adolescents in the US indicates that concurrent tobacco use and poly tobacco use among youth is associated with HTP experimentation [21].

We also found that younger people were more likely to use HTP, in common with the early adoption of other novel products. This is again consistent with more limited previous data from Europe as well as other locations [10]. More educated people were also more likely to report use of HTP. These findings together potentially suggest that the tobacco industry may be trying to build markets by promoting HTP as high profile status symbol products, more akin to tobacco marketing before a range of restrictions. We found that HTP being cool or attractive were a associated with use among younger people, suggesting that the HTP may erode previous gains in tobacco control through both attracting young people into tobacco use but also through renormalisation of the act of tobacco smoking, which HTP mimic. The role of social media in promoting use of HTP as healthy or as part of a luxury or fashion lifestyle substantiate these concerns and deserve regulatory consideration [22].

These results combined with the individual potential health risks of HTP point to the importance of designing and enforcing a robust regulatory framework for HTP, as recommended by the WHO [1]. This includes restrictions on marketing and taxation levels similar to other products [1]. European countries are identified as key markets for HTP by market analysts, and lessons from e-cigarettes are a reminder of the power of the industry to induce or stimulate demand for their products. Analyses of internal documents of Philip Morris International, which is the largest provider of HTP products, have concluded that HTP are viewed as a mechanism to increase the pool of nicotine users and maintain profits [23].

Europe represents a promising market for HTP due to its large population size at around 450 million people and its high proportion of current tobacco smokers at 23% [15]. There is substantial variation between EU countries in the prevalence of HTP use, ranging from 2·8% ever use in France to 14·6% in the Czech Republic. Limited numbers of HTP users in Europe means that we were unable to assess whether the role of factors such as age, sex and education in HTP use was consistent across countries. The variation identified likely points in part to differing local tobacco markets and differential levels of promotion and market penetration, partly driven by differing times of entry of HTP into local markets. These differences underscore the importance of a united regulatory regime at the European level, as highlighted by the current TPD and potentially note the need for more stringent regulation of HTPs under the TPD. Importantly, the issue of HTP should not deter of detract the public health community from maintaining a relentless focus on driving down levels of use and harm from tobacco smoking.

### 4.1 Limitations

While this paper presents up to date analyses of a representative sample of the European population, there are inevitably limitations. First, all data was self-reported meaning that we rely on individuals having an accurate perception of HTP as distinct from other products. Second, the data are cross sectional which precludes us from making any inferences about causality and we present associations only. Importantly, we were unable to assess whether any of the HTP users initiated smoking of combustible tobacco following experimentation with HTP, which would lead to an underestimation of HTP use among never smokers. We were also unable to provide more detailed assessment of the intensity of HTP use, which is likely to be valuable in future research. Third, for the analyses of reasons for using HTPs, the Eurobarometer design means that we were only able to assess these among people using HTP but not e-cigarettes. It is therefore uncertain if these results apply to those using both of these products. Fourth, Eurobarometer does not publish response rates for the survey, although we do present weighted estimates, which account for non-response. Finally, the low percentages of people using HTP, particularly currently and daily, and in certain subgroups means that there are some small numbers and uncertainty around our estimates.

### 4.2 Conclusion

Use of HTP products in Europe was low in 2020, with significant cross-country differences in prevalence, Use was significantly higher among younger people and current or former smokers. Given the serious interests of the tobacco industry in these products, growth is likely and warrants additional regulation when revising EU and national regulatory frameworks.

## Contributors Statement

All authors conceived the paper. FF cleaned the data and performed the analyses. All authors interpreted findings, AL wrote the first draft which all authors revised for intellectual content

## Declaration of interests

Authors have no interests to declare

## Appendix Supplementary materials

Image, application 1
