# Supplementary material for: Prevalence and reasons for use of Heated Tobacco Products (HTP) in Europe: an analysis of Eurobarometer data in 28 countries
Source: Lancet Reg Health Eur. 2021 Jul 14;8:100159. doi: 10.1016/j.lanepe.2021.100159 (PMC8454644; doi:10.1016/j.lanepe.2021.100159)
Supplement: Supplementary file 1 [file mmc1.docx]

**Supplementary Table 1. Analytic sample size by country, Eurobarometer, 2020.**

| **Country** | **Sample size** |
| --- | --- |
| France | 1,001 |
| Austria | 1,008 |
| Belgium | 981 |
| Bulgaria | 1,057 |
| Croatia | 1,019 |
| Cyprus | 505 |
| Czech Republic | 1,027 |
| Denmark | 1,022 |
| Estonia | 1,104 |
| Finland | 1,099 |
| Germany | 1,527 |
| Greece | 1,016 |
| Hungary | 1,058 |
| Ireland | 1,239 |
| Italy | 1,021 |
| Latvia | 1,024 |
| Lithuania | 1,008 |
| Luxembourg | 608 |
| Malta | 502 |
| Netherlands | 1,086 |
| Poland | 1,057 |
| Portugal | 1,061 |
| Romania | 1,103 |
| Slovakia | 1,046 |
| Slovenia | 1,011 |
| Spain | 1,049 |
| Sweden | 998 |
| United Kingdom | 1,063 |
| **TOTAL** | **28,300** |

**Supplementary Table 2. Eurobarometer 2020, analytic sample characteristics.**

|  |  | **N (weighted %)** |
| --- | --- | --- |
| Age | |  |
|  | 55+ years | 12,650 (39.1) |
|  | 40-54 years | 7,248 (25.4) |
|  | 25-39 years | 5,833 (22.9) |
|  | 15-24 years | 2,565 (12.6) |
| Sex | |  |
|  | Female | 15,265 (51.2) |
|  | Male | 13,033 (48.7) |
| Difficulty paying bills | |  |
|  | Never/almost never | 19,642 (70.6) |
|  | From time to time/most of the time | 8,459 (29.4) |
| Education | |  |
|  | Lower secondary or lower | 6,408 (28.6) |
|  | Upper secondary | 12,024 (39.7) |
|  | Tertiary up to bachelor | 5,802 (17.9) |
|  | Masters or above | 4,027 (13.8) |
| Area of residence | |  |
|  | Rural | 9,412 (29.9)) |
|  | Urban | 18,877 (70.1) |
| Smoking | |  |
|  | Never smoker | 15,089 (54.6) |
|  | Current smoker | 6,661 (23.2) |
|  | Former smoker | 6,529 (22.1) |

**Supplementary Table 3. Proportion of heated tobacco products users who concurrently use other nicotine products at least weekly.**

|  | **Among ever HTP users**  **% (95% CI)** | **Among current HTP users**  **% (95% CI)** | **Among daily HTP users**  **% (95% CI)** |
| --- | --- | --- | --- |
|  | N=2,039 | N=373 | N=228 |
| Boxed cigarettes | 40.9 (37.3 – 44.6) | 35.2 (27.5 – 43.7) | 19.4 (12.9 – 28.2) |
| Hand-rolled cigarettes | 15.6 (12.8 – 18.8) | 9.6 (4.9 – 18.0) | 3.7 (1.5 – 8.6) |
| Cigarillos | 1.4 (0.6 – 2.8) | 3.7 (1.0 – 12.3) | 0.3 (0.1 – 1.4) |
| Cigars | 0.6 (0.3 – 1.1) | 0.4 (0.1 – 1.1) | 0.4 (0.1 – 1.5) |
| Pipe | 1.2 (0.5 – 2.8) | 4.7 (1.6 – 13.3) | 2.2 (0.4 – 10.9) |
| E-cigarettes | 6.5 (4.8 – 8.7) | 8.3 (4.4 – 15.0) | 5.8 (2.6 – 12.4) |
| Waterpipe | 5.5 (3.8 – 7.8) | 7.8 (3.7 – 15.9) | 2.0 (0.5 – 8.2) |
| Smokeless tobacco | 3.1 (2.0 – 4.8) | 5.8 (2.3 – 13.7) | 1.4 (0.6 – 3.5) |
| Any combustible tobacco product | 51.3 (47.5 – 55.0) | 41.4 (33.4 – 50.0) | 25.3 (17.6 – 34.9) |
| Any tobacco product | 52.5 (48.7 – 56.2) | 42.5 (34.4 – 51.0) | 26.6 (18.8 – 36.2) |
| Any nicotine product | 56.2 (52.4 – 59.9) | 45.0 (36.8 – 53.5) | 29.5 (21.1 – 39.5) |

Any combustible tobacco products include boxed cigarettes; hand-rolled cigarettes; cigarillos; cigars; pipe; and waterpipe.

Any tobacco products include boxed cigarettes; hand-rolled cigarettes; cigarillos; cigars; pipe; waterpipe; and oral tobacco.

Any nicotine products include boxed cigarettes; hand-rolled cigarettes; cigarillos; cigars; pipe; waterpipe; oral tobacco; and e-cigarettes containing nicotine.

**Supplementary Table 4. Reasons to use heated tobacco products in 28 European countries, 2020.**

|  | Weighted % (95% CI) |
| --- | --- |
| You believed that these products were less harmful than smoking tobacco products | 39.5 (23.4 – 47.0) |
| Your friends used heated tobacco products | 28.4 (21.7 – 36.2) |
| To stop or reduce tobacco smoking | 28.2 (22.2 – 35.1) |
| You liked the flavours of heated tobacco products | 22.1 (16.4 – 29.0) |
| You could consume tobacco in places where tobacco smoking was not allowed | 18.9 (14.1 – 24.9) |
| They were cool or attractive | 17.7 (12.7 – 24.1) |
| They were cheaper than other tobacco products | 14.2 (9.6 – 20.5) |

Note: Percentages shown among respondents who ‘use or used heated tobacco products’, but not e-cigarettes (n=460).
